# An Interactive Simulation to Change Outcome Expectancies and Intentions in Adults With Type 2 Diabetes: Within-Subjects Experiment

**DOI:** 10.2196/diabetes.8069

**Published:** 2018-01-09

**Authors:** Bryan Gibson, Leah Yingling, Alisa Bednarchuk, Ashwini Janamatti, Ingrid Oakley-Girvan, Nancy Allen

**Affiliations:** 1 Department of Biomedical Informatics University of Utah Salt Lake City, UT United States; 2 Henrietta Schmoll School of Health St. Catherine University St Paul, MN United States; 3 School of Computing University of Utah Salt Lake City, UT United States; 4 Cancer Prevention Institute of California Fremont, CA United States; 5 Stanford Cancer Institute Stanford University Palo Alto, CA United States; 6 Canary Center at Stanford for Cancer Early Detection Stanford University Palo Alto, CA United States; 7 School of Nursing University of Utah Salt Lake City, UT United States

**Keywords:** diabetes mellitus, type 2, computer simulation, beliefs, intention

## Abstract

**Background:**

Computerized simulations are underutilized to educate or motivate patients with chronic disease.

**Objective:**

The aim of this study was to test the efficacy of an interactive, personalized simulation that demonstrates the acute effect of physical activity on blood glucose. Our goal was to test its effects on physical activity-related outcome expectancies and behavioral intentions among adults with type 2 diabetes mellitus (T2DM).

**Methods:**

In this within-subjects experiment, potential participants were emailed a link to the study website and directed through 7 tasks: (1) consent; (2) demographics, baseline intentions, and self-reported walking; (3) orientation to the diurnal glucose curve; (4) baseline outcome expectancy, measured by a novel drawing task in which participants use their mouse to draw the expected difference in the diurnal glucose curve if they had walked; (5) interactive simulation; (6) postsimulation outcome expectancy measured by a second drawing task; and (7) final measures of intentions and impressions of the website. To test our primary hypothesis that participants’ outcome expectancies regarding walking would shift toward the outcome presented in the interactive simulation, we used a paired *t* test to compare the difference of differences between the change in area under the curve in the simulation and participants’ two drawings. To test whether intentions to walk increased, we used paired *t* tests. To assess the intervention’s usability, we collected both quantitative and qualitative data on participants’ perceptions of the drawing tasks and simulation.

**Results:**

A total of 2019 individuals visited the website and 1335 (566 males, 765 females, and 4 others) provided complete data. Participants were largely late middle-aged (mean=59.8 years; standard deviation=10.5), female 56.55% (755/1335), Caucasian 77.45% (1034/1335), lower income 64.04% (855/1335) *t*_1334_=3.4, *P* ≤.001). Our second hypothesis, that participants’ intentions to walk in the coming week would increase, was also supported; general intention (mean difference=0.31/7, *t*_1001_=10.8, *P*<.001) and minutes of walking last week versus planned for coming week (mean difference=33.5 min, *t*_1334_=13.2, *P*<.001) both increased. Finally, an examination of qualitative feedback and drawing task data suggested that some participants had difficulty understanding the website. This led to a post-hoc subset analysis. In this analysis, effects for our hypothesis regarding outcome expectancies were markedly stronger, suggesting that further work is needed to determine moderators of the efficacy of this simulation.

**Conclusions:**

A novel interactive simulation is efficacious in changing the outcome expectancies and behavioral intentions of adults with T2DM. We discuss applications of our results to the design of mobile health (mHealth) interventions.

## Introduction

### Background

Type 2 diabetes mellitus (T2DM) affects 29 million people in the United States and is associated with significant morbidity and early mortality [1]. Regular physical activity is considered one of the cornerstones of diabetes self-management [2] and has been shown to improve glycemic control [3], reduce blood pressure [4], and improve cardiorespiratory fitness in individuals with T2DM [5]. These intermediate outcomes have been associated with reduced diabetes-related morbidity and mortality [6]. Despite these benefits, most people with T2DM do not perform recommended amounts of physical activity [7].

By virtue of their potential for scalability and personalization, Web-based interventions have great potential to facilitate self-management in individuals with diabetes. However, to date, most interventions have demonstrated only small to moderate effects on self-management behaviors [8]. One reason for this may be that most interventions have used only a limited number and palette of behavior change techniques (BCTs) [9] (the smallest observable and replicable *active ingredient* in a behavioral intervention [10]). Several prominent theorists have proposed that, before deploying complex multicomponent mobile health (mHealth) interventions, designers and investigators should first demonstrate that each of the interventions’ components have demonstrated efficacy [11,12].

### Objective

In this study, we sought to test the efficacy of a novel BCT; an interactive Web-based simulation that demonstrates the immediate positive consequences of behavior change. The power of an interactive simulation is that it allows the user to experiment with possible actions and learn by vicariously experiencing the outcomes of those actions [13]. Simulations are now regularly used for the training of health care providers (HCPs) [14], but little research has addressed their use as an education and behavior change tool for patients.

Outcome expectancies are an individual’s belief regarding the likely outcome of a given behavior (eg, what will happen to my blood sugar if I walk) [15]. Prior work has shown that outcome expectancies are related to self-care behaviors in individuals with T2DM [16] and that individuals with T2DM generally have low outcome expectancies regarding the effect of exercise [17]. Outcome expectancies are usually measured using Likert type scales (eg, “walking will improve my blood sugar control” strongly disagree—strongly agree). In this study, we used an electronic drawing task to measure participants’ outcome expectancies. This electronic method allowed us to directly compare people’s beliefs with the outcome presented by the simulation using area under the curve (AUC).

### Prior Related Work

In prior work, we used daily glucose curves to change outcome expectancies regarding the immediate glycemic effects of exercise in adults with T2DM [18,19]. In this study, we sought to build on and improve upon our prior work in several ways. First, in our earlier work, the demonstration of the immediate positive consequences of behavior change was combined with other BCTs (eg, demonstrating negative consequences of failure to change behavior, guiding the individuals in action planning, and providing social support modeling the target behavior). In this study, we deliberately isolated the demonstration of the immediate positive consequences of behavior change to estimate its stand-alone efficacy. Second, in our prior work, the demonstration of the immediate positive consequences of behavior change reflected the average effect for an average person. Because the true effect of physical activity on blood glucose varies significantly across individuals [20], a personalized estimate of the effect is preferable and more accurate. In this study, we took a first step toward true personalization by presenting the effect for someone with similar blood glucose control (hemoglobin A_1c_, HbA_1c_) as the participant. Finally, because our prior work involved in-person interventions, the sample sizes were necessarily small and limited in diversity. In this study, we made a concerted effort to recruit a large and diverse sample of adults with T2DM.

### Hypotheses

We hypothesized (1) that use of the simulation would shift users’ outcome expectancies toward the outcome presented in the simulation and (2) that use of the simulation would lead to an increase in intentions to be physically active.

## Methods

### Human Subjects Protection

This study was reviewed and approved by the University of Utah Institutional Review Board.

### Recruitment

Recruitment for this study was done simultaneously with a parallel study (manuscript in process) conducted with HCPs who treat individuals with T2DM. For both studies, we recruited participants via email.

An email invitation was disseminated directly to patients via the email list of clients of Alliance Health; a national provider of diabetic testing supplies.

An email invitation was also sent to the following groups of clinicians: a listserv of providers and diabetes educators from the Utah Department of Health; listservs of faculty and students at the University of Utah schools of medicine, nursing, and physical therapy; faculty and students of New York University schools of nursing and medicine; colleagues at Stanford University and at the Cancer Prevention Institute of California; and several community collaborators. The email invitation included the statement “please feel free to share this link with patients with type 2 diabetes and clinician colleagues.” In this way, we intended to indirectly invite patients with T2DM. This snowball sampling approach aimed to recruit as geographically, ethnically, and socioeconomically diverse a sample as possible. Study participation was incentivized by including participants in a lottery for one of five US $100 gift cards.

### Screening

After opening the website, participants self-sorted by clicking one of three statements (hyperlinks):

- “I am a person with Type 2 Diabetes” (participant directed to study website)
- “I am a healthcare provider or trainee who treats patients with Type 2 Diabetes” (participant directed to provider-facing website)
- “I am neither a person with Type 2 Diabetes nor a Healthcare provider” (participant thanked and dismissed)

### Study Website

Participants completed all study tasks during a single session on the study website.

**Figure 1:**
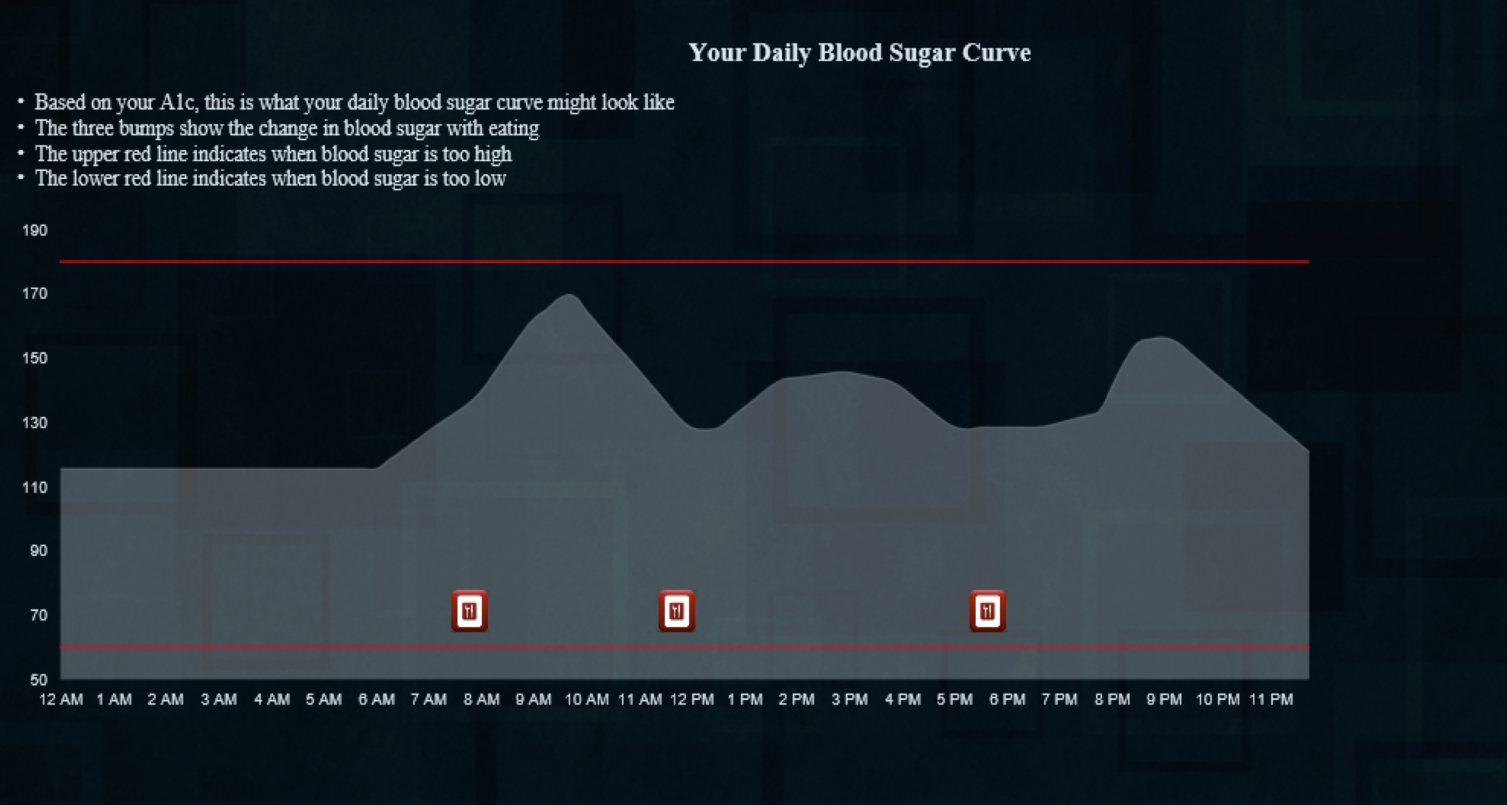
Orientation to the diurnal glucose curve.

**Figure 2:**
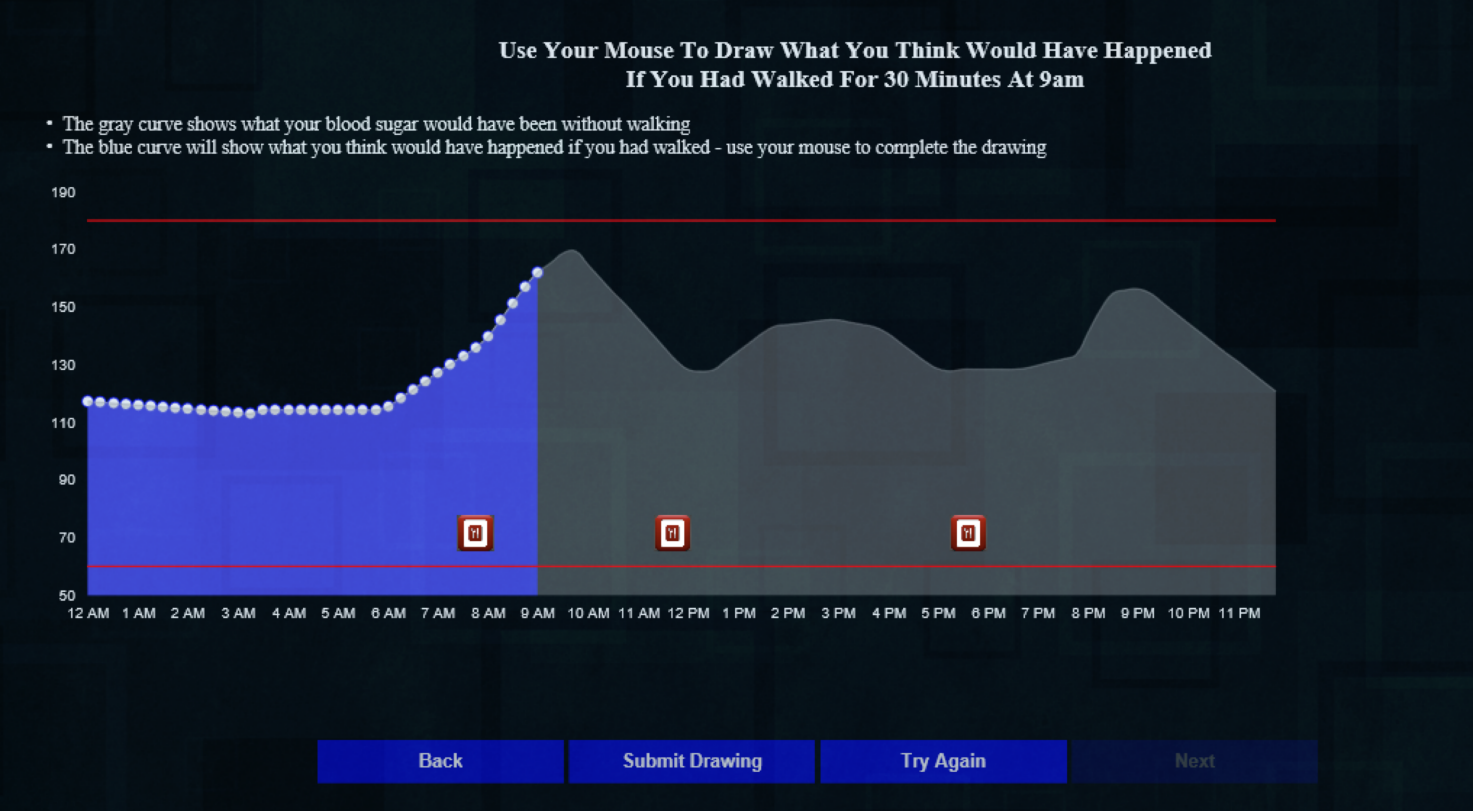
Drawing task.

**Figure 3:**
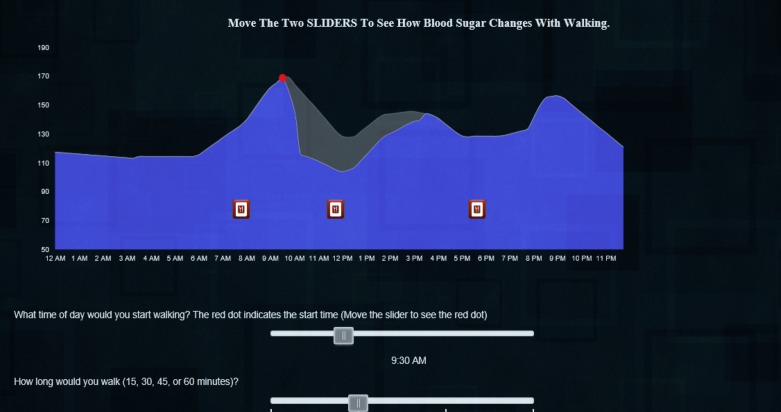
Screenshot of simulation.

The study website leads participants through seven tasks (in fixed order):

1. Consent cover letter
2. Participant characteristics, past week walking, and presimulation intentions to be active. Participants completed 13 questions regarding demographics, diabetes-specific data (eg, self-reported HbA_1c_ and treatments), self-reported days and minutes of walking in the last week, and general intentions to be active (7-point Likert scale).
3. Orientation to the diurnal glucose curve (Figure 1). This task displayed a static graph showing a diurnal glucose curve with icons indicating when the person ate and some brief, simple language to orient individuals naïve to this type of graph. The glucose values in this graph, the subsequent drawing task, and the interactive simulation are based on prior work in which we developed “average” daily glucose curves for each HbA_1c_ value from 5.9 to 10.1 (in increments of 0.1) [21]. Using these curves allowed us to personalize the simulation, to some degree, for each participant.
4. Presimulation outcome expectancies. First drawing—using their cursor, participants drew what they believed the glucose curve would have looked like had they walked for 30 min at 9 AM (Figure 2).
5. Simulation. Participants could move two sliders, one to change the time of day and the second to vary the duration of exercise to see what effect walking at different times of day and for different durations of 15, 30, 45, or 60 min might have on their glucose curve (Figure 3). To calculate the effect of exercise on glucose, we estimated the glucose value 30 min after exercise using a predictive model we developed in prior work, [20] and conservatively estimated that glucose would return to non-exercise levels over the following 6 hours [22].
6. Postsimulation outcome expectancies. Second drawing—After exploring the simulation, participants again drew what they believed the glucose curve would have looked like had they walked for 30 min at 9 AM. The interface for this drawing task was identical to the first (Figure 2).
7. Intentions to be active and feedback on website and study. On the final tab of the website, participants indicated their intentions to be active: general intentions to be active on a 7-point Likert scale and numeric values for planned minutes and days of walking in the coming week. They also rated the website's utility and informativeness (7-point Likert scales to rate how useful [“This website was useful” 1=strongly agree to 7=strongly disagree] and informative [“This website was *not* informative” 1=strongly agree to 7=strongly disagree]). Finally, a free text box titled “Please provide any feedback you have on this website or study” allowed participants to optionally provide qualitative feedback.

### Analysis

To calculate the AUC for the “no walking” curve (Figure 1), we took the vector of values for the curve corresponding to the participant’s self-reported HbA_1c_ and multiplied by 15 to get the total AUC in milligram/deciliter×minutes (this was necessary because the blood glucose values for the curve represent values in increments of 15 min).

To calculate the AUC for the drawing tasks, we first combined the vector of glucose values for the “no walking” curve (Figure 1) from 12 AM to 9 AM with the values that the participant drew. In cases where participants’ drawings ended before the end of the day, we interpolated values between their last drawn point and the value at the end of the day (12 midnight) from the “no walking” curve. We then multiplied that vector by 15 (for our 15-min intervals) to get the total AUC in milligram/deciliter×minutes.

We used a similar process to calculate the AUC for the interactive simulation. In this case, we combined the vector of glucose values for the “no walking” curve (Figure 1) from 12 AM to 9 AM with the estimated postexercise glucose, (based on our predictive model) and interpolated a proportional return to the value of the “no walking” curve at 3 PM (6 hours after the start of walking).

### Analysis for Hypothesis 1: Participants’ Outcome Expectancies Will Shift Toward the Outcome Presented in the Simulation

We calculated the differences in the AUC between the participants’ counterfactual (no walking) glucose curve and their two drawings (presimulation and postsimulation). We then calculated the differences between these drawn beliefs (outcome expectancies) and the simulation. To determine if outcome expectancies changed as a result of using the simulation, we compared these differences using a paired *t* test.

### Analysis for Hypothesis 2: Use of Simulation Will Increase Intentions

To test whether intentions to walk in the coming week changed, we used paired *t* tests. For the minutes of walking/week, we simply compared the participants’ reported minutes of walking in the last week with their planned minutes of walking in the coming week and their pre- and postsimulation rating. To test whether ratings on a 7-point Likert scale to the statement “I intend to walk in the coming week” changed, we also used a paired *t* test.

To address missing data, we used *t* tests on only the complete cases (discarding individuals with missing data) and on two types of imputed data: first we replaced missing points data with mean values for postsimulation intentions, and second, we replaced missing values with the individuals’ presimulation intentions. We report the most conservative of these findings.

### Participants’ Perceptions of the Website

We calculated the mean and standard deviation (SD) for participants’ ratings of the website’s informativeness and usefulness.

Next, we conducted standard qualitative analyses of participants’ free text feedback. Three investigators (BG, LY, and VD) reviewed this feedback and independently coded participant comments according to eight categories determined by the coders to encompass feedback relevant to our website design and to future research: positive feedback (on content or functionality), negative feedback on content, negative feedback on understandability, negative feedback on the relevance of the site’s content to the participant, negative comment on usability, spontaneous mention of barriers to physical activity, suggestion for additional content or functionality, and miscellaneous comments. Participant comments could be associated with more than one code. After initial coding, the three investigators reviewed initial coding and reconciled until they reached >85% agreement for each quote.

### Subset Analysis

The results of our primary quantitative and qualitative analysis led us to perform a post-hoc subset analysis looking at the effects of the intervention, in which we removed individuals who either self-reported a lack of understanding of the drawing tasks or simulation or whose drawings were extreme outliers.

### Post-Hoc Analysis

Finally, we created a set of four post-hoc regression models to determine if the baseline measures we had collected on participants were associated with their baseline outcome expectancies or with intervention efficacy—changes in outcome expectancy, changes in planned minutes in walking/week, or changes in intentions to walk.

## Results

### Participants

Of the 2019 unique individuals who visited the website, 1335 (566 males, 765 females, and 4 others) provided complete data. As described in Tables 1 and 2, participants were predominately late middle-aged (mean=59.8 years, SD=10.5), female 56.55% (755/1335), Caucasian 77.45% (1034/1335), lower income 64.04% (855/1335) <US $40,000/year annual household income), and geographically diverse (52 US states and territories).

Participants were nearly equally split between treatment with oral medications 48.76% (651/1335) and injectable medications 44.57% (595/1335); most had previously attended diabetes education 82.39% (1100/1335) and most reported generally well-controlled glucose (mean HbA_1c_ 7.3%, SD=1.2). More than half 57.83% (772/1335) reported walking for exercise in the previous week.

### Hypothesis 1: Participants’ Outcome Expectancies Will Shift Toward the Outcome Presented in the Simulation

#### Presimulation Outcome Expectancies

Compared with the simulation, which was conservative in its estimate of the expected effect (mean decrease in AUC of 5712 mg/dl×min, SD=2033 mg/dl×min), most individuals’ presimulation outcome expectancies were overly positive (mean decrease in AUC of 12,265 mg/dl×min, SD=20,253).

#### Postsimulation Expectancies

As hypothesized, participants’ postsimulation outcome expectancies shifted toward the outcomes presented by the simulation; mean decrease in AUC of 10,582 mg/dl×min, SD=19,117 mg/dl×min.

A paired *t* test comparing the difference of differences between the first drawing and the simulation (mean difference 6553 mg/dl×min, SD=19,230) and the second drawing and the simulation (mean difference 4869 mg/dl×min, SD=18,270) indicated a statistically significant shift in outcome expectancies toward the outcome presented by the simulation (mean of the differences=1683.4, *t*_1334_=3.4, *P* ≤.001).

### Hypothesis 2: Use of Simulation Will Increase Intentions

#### Pre-and Postsimulation Intentions to Be Active

Our second hypothesis, that participants’ intentions to walk in the coming week would increase, was supported in both measures; general intention increased (mean difference=0.31, *t*_1001_=4.53, *P*<.001).

Similarly, when assessing whether minutes of walking planned for the coming week increased over minutes of walking reported in the past week, the intervention had a positive effect (mean difference=33.5 min, *t*_1334_=13.2, *P*<.001).

Table 3 presents the presimulation and postsimulation means and standard deviations for the measures for these two hypotheses.

### Feedback on Website

Multimedia Appendix 1 contains the results of analysis of responses to the statements “This website was informative” and “This website was *not* useful” (1=strongly agree to 7=strongly disagree), as well as the result of our qualitative analysis of individuals free text feedback on the study or website.

### Subset Analysis

We conducted a subset analysis to determine whether our findings regarding changes in outcome expectancies and intentions held true after excluding individuals for whom the drawing task may not have accurately reflected their beliefs (because of suboptimal understanding) or who reported significant difficulty understanding the simulation.

This yielded two categories of potential individuals to exclude (1) participants whose first and second drawings were marked outliers from the expected effect and (2) individuals who directly commented in the final comments text box that they did not understand either the curves or the simulation. These latter groups of individuals were excluded only, if, on a subsequent independent review, all three coders agreed to exclude.

The resulting subset included 1194 individuals. Table 4 summarizes the mean and SDs for outcome expectancies, general intentions, and minutes walking (reported vs planned) for this group of participants. From this table, it is clear that for intentions, the results for this subset of participants are nearly identical to the full group; however, for outcome expectancies, the efficacy of the simulation is stronger, and individuals’ postsimulation beliefs are on average almost identical to those presented in the simulation (mean decrease in AUC of 5712 mg/dl×min, SD=2033 mg/dl×min).

### Post-Hoc Analysis: Were Baseline Outcome Expectancies or Intervention Efficacy Associated With Demographics or Treatment Class?

Multimedia Appendix 1 contains the results of the post-hoc regression models we created to determine whether demographics (sex and age) or clinical variables (treatment type and HbA_1c_) were associated with either baseline outcome expectancy or intervention efficacy: changes in outcome expectancy, planned walking minutes /week, or behavioral intentions to walk.

**Table 1:** Continuous demographics.

Characteristic	Mean (SD^a^) or n (%)
Age (in years), mean (SD)	59.9 (10.5)
Hemoglobin A_1c_ level, n (%)	7.3 (1.2)

^a^SD: standard deviation.

**Table 2:** Categorical demographics.

Characteristic		n (%)
**Sex**		
	Female	755 (56.55)
	Male	566 (42.39)
	Other	4 (0.003)
**Race**		
	Caucasian	1034 (77.45)
	Hispanic	63 (4.72)
	Asian or South Asian	27 (2.02)
	African American	124 (9.28)
	American Indian or Native American	12 (0.89)
	Native Hawaiian or Pacific Islander	3 (0.22)
	Multiethnic	15 (1.12)
**Annual income (in US dollars)**		
	<20,000	582 (43.59)
	20,000-39,999	273 (20.44)
	40,000-59,999	183 (13.70)
	60,000-79,999	123 (9.21)
	80,000-99,999	174 (13.03)
**Region of residence**		
	Northeast	184 (13.78)
	South	503 (37.67)
	Midwest	326 (24.44)
	West	317 (23.74)
	US territories	5 (0.37)
**Health insurance coverage**		
	Yes	1335 (100.00)
	No	0 (0)
**Primary care provider established**		
	Yes	1335 (100.00)
	No	0 (0)
**Primary language**		
	English	1335 (100.00)
	Other	0 (0)
**Previous diabetes education**		
	Yes	1100 (82.39)
	No	235 (17.60)
**Diabetes treatment type**		
	Injectable medications	595 (44.57)
	Oral medications	651 (48.76)
	Diet and exercise	81 (6.06)
**Walked for exercise last week (presimulation assessment)**		
	Yes	772 (57.82)
	No	563 (42.17)

**Table 3:** Behavioral intentions and outcome expectancies before and after simulation.

Outcome measure	Presimulation	Postsimulation
Outcome expectancy (glucose levels)	12,265 mg/dl×min (20,253)	10,582 mg/dl×min (19,117)
Intentions to walk in next week^a^, mean (SD^b^)	5.16 (1.8)	5.47 (1.6)
Minutes walking, mean (SD)	67.1 (88.0) in last week	100.5 (100.4) planned

^a^“I intend to walk in the coming week” rated on a 7-point scale from 1=“strongly disagree” to 7=“strongly agree.”

^b^SD: standard deviation.

**Table 4:** Intentions before and after simulation (subset of 1194 participants).

Outcome measure	Presimulation	Postsimulation	*t* score, *P* value
Intentions to walk in next week^a^, mean (SD^b^)	5.2 (1.8)	5.46 (1.7)	9.7, <.001
Minutes walking, mean (SD)	68.6 (89.4) in last week	98.7 (100.4) planned	11.2, <.001
Outcome expectancy (glucose levels)	7852 mg/dl×min (15,284)	5890 mg/dl×min (12,536)	4.2, <.001

^a^“I intend to walk in the coming week” rated on a 7-point scale from 1=“strongly disagree” to 7=“strongly agree.”

^b^SD: standard deviation.

## Discussion

### Principal Findings

This study tested whether an interactive Web-based simulation would change participants’ outcome expectancies regarding the acute effects of behavior change and whether use of the simulation would also be associated with an increase in participants’ intentions to engage in the behavior. Specifically, we conducted a within-subjects experiment to determine if an interactive simulation that shows the acute effects of physical activity on the diurnal glucose curve would affect outcome expectancies and intentions to be active in adults with T2DM. We found that use of the simulation shifted individuals’ outcome expectancies (measured by a novel drawing task) toward the outcome presented by the simulation and that users’ general intentions to be active and their planned minutes of walk in the coming week both increased. We are encouraged by these results but also believe that they suggest the need for several areas of further work, which we discuss below.

The results of this study are in line with our prior work, which found that using glucose curves to demonstrate the acute positive effects of physical activity improves outcome expectancies, self-efficacy, behavioral intentions to be active in the future, and activity in the short term [18,19,23]. This study tested this simulation in isolation from other BCTs (in contrast to our prior work that employed many BCTs) and recruited a large and diverse sample. Taken together, we believe these studies provide evidence that demonstrating to adults with T2DM the acute positive effects of behavior change is efficacious and should be included in more behavioral interventions.

Despite our positive finding on the efficacy of the intervention in increasing behavioral intentions, our expected mechanism of action was not supported. We expected that participants would underestimate the effect of physical activity on blood glucose in the first drawing task, and then, after they used the simulation, participants’ outcome expectancy would become more positive. Consistent with several models of health behavior change [15], we expected this increase in positive outcome expectancies would lead to greater intentions to be active. This is not what we found. On average, participants overestimated the effect of exercise in the first drawing task, and the simulation shifted toward the outcome presented but in the opposite direction expected (becoming less positive instead of more). Despite this decrease in outcome expectancies, participants’ intentions to be physically active increased. We believe the most likely explanation for this finding is that in the first drawing, participants were uncertain about their belief (the diurnal glucose curve is unfamiliar to most individuals with T2DM and drawing their expectations of the effects of behavior on the curve is novel), and the first drawing was a “guestimate.” When the simulation confirmed the positive effects of physical activity on glucose, participants’ certainty in the positivity of the effect increased, and therefore, their intentions to be active increased. This hypothesis is supported by research from educational psychology showing that certainty is a moderator of the relationship between students’ expectancies and task performance [24], and that certainty influences the efficacy of persuasive messages [25] and moderates the relationship between attitudes and behaviors [26]. It is worth noting that in searching the literature related to certainty and beliefs, we did not find any studies that measured certainty related to health-related outcome expectancies. Therefore, in addition to investigating this hypothesis for our own work, we suggest that it may be worthwhile to measure participants’ certainty regarding their beliefs more broadly in health-related studies.

### Strengths

This study has several strengths. First, our novel electronic drawing task as a measure of individuals’ outcome expectancies allows for a finer grained quantitative representation of the individual’s belief (AUC). We believe this method warrants further investigation. Future analyses using this drawing method could address questions such as: are measures other than AUC (eg, the coefficient of variation of postexercise glucose or the total AUC under 70 mg/dl) associated with intentions to be active? In addition, this drawing method could be used to understand patient's beliefs about other measures that are relevant to patients’ self-management of chronic disease, including both those for which a “ground truth” is available (eg, ambulatory blood pressure and heart rate) and those that are entirely subjective (eg, mood and pain).

A second strength of this study is that we isolated the effect of our BCT to estimate its efficacy. We believe that more studies in the electronic health or mHealth arena need to take this approach either through simple isolated experiments such as this one or a fractional factorial design to test multiple potential components at once [27]. The value of this approach is that when intervention designers set out to develop complex interventions, they can combine components that are known to be efficacious. A final strength of this study is the large and diverse sample we were able to recruit via our email snowball sampling technique.

### Limitations

Study results should be interpreted in light of the following limitations. First, some participants reported difficulties in completing the drawing tasks and using the simulation. We are currently redesigning the simulation to address the usability issues uncovered in this study. Second, to minimize participant burden, we left out potential moderators of the efficacy of the intervention. For example, the fact that some individuals (eg, those we excluded for the subset analysis) expressed extremely positive or negative outcome expectancies could be attributed to low health literacy [28] or numeracy [29], or it might be that those drawings accurately reflect the individuals’ beliefs. To address this question, future experiments should measure these potential moderators of simulation efficacy. Third, our primary outcome of behavioral intentions to be physically active might be biased because of social desirability. Although some prior work has found evidence for this bias, the effect was small [30]. In addition, a large body of evidence has found that changes in intentions lead to changes in behavior [31]. Finally, some participants commented that they did not trust the simulation because they did not think it was personally relevant. To address this issue, in future work, we might make areas of uncertainty more explicit (eg, show the 95% CI around the simulated glucose curve or the predicted effect). Future work might also maximize the personal relevance of the simulation by integrating patient-specific data (eg, individuals’ continuous glucose monitoring curve and accelerometry data).

### Conclusions

Our Web-based, interactive simulation shifted outcome expectancies and increased participants’ intentions to be physically active. Further work will examine the effect of the simulation on objectively measured behavior. We suggest that simulations that demonstrate the acute positive effects of behavior change might generalize to the promotion of other health behaviors and other chronic diseases.

## Appendix

Multimedia Appendix 1 Supplementary file 1.

## Abbreviations

AUC: area under the curve
BCT: behavior change technique
HbA_1c_: hemoglobin A_1c_
HCP: health care provider
mHealth: mobile health
SD: standard deviation
T2DM: type 2 diabetes mellitus
