# Supplementary material for: An Interactive Simulation to Change Outcome Expectancies and Intentions in Adults With Type 2 Diabetes: Within-Subjects Experiment
Source: JMIR Diabetes. 2018 Jan 9;3(1):e2. doi: 10.2196/diabetes.8069 (PMC6238889; doi:10.2196/diabetes.8069)
Supplement: Multimedia Appendix 1 [file diabetes_v3i1e2_app1.pdf]

**Supplemental File 1.**

## Feedback on website

In response to the statements “This website was informative” and “This website was *not* useful” (1- strongly agree to 7- strongly disagree), participants were generally positive. On average, participants felt that the website was informative (mean=2.9/7; SD 1.7) and disagreed that the site was *not* useful (mean= 5.2/7, SD=1.8).

Finally, 394 participants provided qualitative feedback in the “Please provide any feedback you have on this website or study” text box. Here we report the coding for quotes in which all three coders agreed. Out of 394 comments, 112 were coded as positive, for example: “Definitely an eye-opener. Will get out and walk as much as I can!” Twenty- five comments were coded as constructive/negative feedback of a general nature on the content of the website or study such as “an interesting study, but not really sure how much it would help.” Forty-two comments were coded as concern about the personal relevance of the study, for example “The numbers were way off. My sugar is not that high.” Forty-eight comments were related to comments on the content or understandability of the website for example “not sure what we are supposed to get out of the website?” Twenty-three comments provided constructive feedback on the usability of the website, for example, “Many of us use smart phones and tablets, it would be nice if the survey was user friendly for those devices.” Sixty-eight comments addressed spontaneous mentions of barriers to physical activity (particularly walking), for example, “This does not take into account bad knees and hips, walking is painful.” Forty-four comments addressed desired additional functionality “Good tool - what would happen if you walked multiple time during the day for 15 minutes each time?” A final group of forty-eight comments were coded as miscellaneous, for example “I always feel better after I walk, more energetic and calmer, happier.”

## Post- Hoc analysis: were baseline outcome expectancies or intervention efficacy associated with demographics or treatment class?

In this post-hoc analysis we created four the regression models to determine whether demographics (sex, age), or clinical variables (treatment type, HbA1c) were associated with either baseline outcome expectancy or intervention efficacy: change in outcome expectancy, planned walking minutes /week or behavioral intentions to walk.

As can be seen in Table 2. Males had slightly more negative baseline outcome expectancies than females and the higher a participants’ HbA1c was, the more positive their baseline outcome expectancies were. This effect would be expected because increased HbA1c leads to increased area under the daily glucose curve. Treatment class and age were not significantly associated with baseline outcome expectancies

**Table 2. Linear model regressing baseline outcome expectancies on demographic variables, HbA1c and treatment class**

| Variable | Coefficient (Beta) | Standard Error | T value | P value |
|---|---|---|---|---|
| Oral medication | 1,674.8 | 1703.4 | 0.9 | 0.33 |
| Injectable medication | 1,282.5 | 1764.4 | 0.7 | 0.46 |
| Hemoglobin A1c | 5,291.3 | 443.4 | 11.9 | <0.001 *** |
| Sex | -1,966.6 | 850.3 | -2.3 | 0.02 * |

| | | | | |
|---|---|---|---|---|
| Age | 38.2 | 41.2 | 0.9 | 0.4 |

**Model Adjusted R-Sq.= 0.117**

Regarding intervention efficacy, as Table 3 shows increased HbA1c was associated larger decreases in outcome expectancy after using the simulation. This was likely because these individual had more room (area under the curve) to err on the side of positive outcome expectancies at baseline.

**Table 3. Coefficients of linear model regressing change in outcome expectancies on demographic variables, HbA1c and treatment class**

| Variable | Coefficient (Beta) | Standard Error | T value | P value |
|---|---|---|---|---|
| Oral medication | -244.5 | 1840.8 | -0.1 | 0.9 |
| Injectable medication | 1266.0 | 1906.4 | 0.6 | 0.46 |
| Hemoglobin A1c | -3661.2 | 482.0 | -7.4 | <0.001*** |
| Male | 705.3 | 918.8 | 0. 7 | 0.44 |
| Age | -50.5 | 44.6 | -1.1 | 0.25 |

**Model adjusted R-squared: 0.044**

Regarding changes in intentions to walk in the coming week. Table 4 shows that only Hemoglobin A1c was associated with increases to walk in the coming week when assessed as minutes /week

**Table 3. Coefficients of Linear model regressing changes in intentions to walk (minutes/ week) on demographic variables, HbA1c and treatment class**

| Variable | Coefficient (Beta) | Standard Error | T value | P value |
|---|---|---|---|---|
| Oral medication | 3.0 | 11.0 | 0.28 | 0.78 |
| Injectable medication | 4.1 | 11.4 | 0.35 | 0.72 |
| Hemoglobin A1c | 6.3 | 2.87 | 2.2 | 0.029 * |
| Male | -0.73 | 5.5 | -0.13 | 0.89 |
| Age | -0.18 | 0.26 | -0.65 | 0.51 |

**Adjusted R-Sq.=0.001**

Finally table 4 shows that neither demographics, HbA1c or treatment class was associated with changes in intentions measure on a 7 point Likert scale.

**Table 4. Coefficients of Linear model regressing changes in intentions to walk (7 point Likert scale) on demographic variables, HbA1c and treatment class**

| Variable | Coefficient (Beta) | Standard Error | T value | P value |
| --- | --- | --- | --- | --- |
| Oral medication | -0.09 | 0.17 | -1.8 | 0.06 |
| Injectable medication | -0.04 | 0.18 | -0.52 | 0.60 |
| Hemoglobin A1c | 0.015 | 0.04 | 0.33 | 0.74 |
| Sex | -0.07 | 0.084 | -0.85 | 0.39 |
| Age | 0.0008 | 0.004 | 0.20 | 0.84 |

**Model Adjusted R-Sq.=0.005**
